# ^68^Ga-pentixafor PET/CT shows better diagnostic performance than ^18^F-FDG PET/CT in chronic lymphocytic leukemia/small lymphocytic lymphoma: a case report and review of literature

**DOI:** 10.3389/fonc.2026.1699568

**Published:** 2026-04-02

**Authors:** Detao Wu, Ranliang Hua, Ze Mao, Yujing Hu, Jingjie Zhang, Congna Tian, Lu Zheng, Xinchao Zhang, Yanzhu Bian

**Affiliations:** 1 School of Clinical Medical, North China University of Science and Technology, Tangshan, China; 2 Department of Nuclear Medicine, Hebei General Hospital, Shijiazhuang, Hebei, China; 3 Department of Emergency, Hebei General Hospital, Shijiazhuang, Hebei, China; 4 Hebei Provincial Key Laboratory of Cerebral Networks and Cognitive Disorders, Hebei General Hospital, Shijiazhuang, China

**Keywords:** case report, CLL/SLL, FDG, pentixafor, PET/CT

## Abstract

Chronic lymphocytic leukemia/small lymphocytic lymphoma (CLL/SLL) is an indolent B-cell lymphoma for which current imaging modalities offer limited diagnostic value. This article reports a comparison of the imaging features of ^18^F-FDG and ^68^Ga-pentixafor in this disease and reviews the relevant literature. A 60-year-old woman presented with a 6-month history of fever and scattered systemic lymphadenopathy. The patient underwent both ^18^F-FDG and ^68^Ga-pentixafor PET/CT imaging within a 2-day period. ^68^Ga-pentixafor PET/CT identified significantly increased metabolic activity in enlarged lymph nodes, the spleen, and bone marrow. Immunohistochemical staining demonstrated a phenotypic profile positive for CD5, CD20, and CD23; showed focal positivity for cyclin D1; and was negative for CD10, BCL-6, and SOX11, findings consistent with the diagnosis of CLL/SLL. Compared with ^18^F-FDG, ^68^Ga-pentixafor PET/CT detected more lesions with significantly higher tracer uptake. This case suggests that, for patients with CLL/SLL, ^68^Ga-pentixafor PET/CT demonstrates significantly superior performance in evaluating the tumor burden and delineating the disease extent compared with ^18^F-FDG PET/CT.

## Introduction

Chronic lymphocytic leukemia (CLL) and small lymphocytic lymphoma (SLL), indolent B-cell lymphomas, represent the same disease entity with different clinical presentations. Accurate assessment of disease involvement is critical for clinical management. While CT and ^18^F-FDG PET/CT are currently used for lesion localization and staging, their utility is limited by the generally low or absent FDG avidity of these tumors (1). The C–X–C chemokine receptor type 4 (CXCR4), a G protein-coupled receptor (GPCR), is highly expressed on tumor cells (2). ^68^Ga-pentixafor, a high-affinity ligand for CXCR4, enables specific receptor targeting. PET/CT with ^68^Ga-pentixafor visualizes *in vivo* CXCR4 expression, offering a promising tool for staging and clinical decision-making in CLL/SLL (3). We present a case demonstrating intense ^68^Ga-pentixafor uptake across multiple enlarged lymph nodes, the spleen, and bone marrow. This highlights the superior diagnostic value of this technique in precisely defining the disease extent, particularly in the context of low ^18^F-FDG avidity.

## Case presentation

A 60-year-old female patient was admitted due to intermittent cough, sore throat, and fever (peak temperature, 39.4°C) for 6 months without obvious cause. Chest CT revealed multiple enlarged lymph nodes in the mediastinum, bilateral hila, retroperitoneum, bilateral axillae, and supraclavicular regions. To further clarify the diagnosis, the patient underwent ^18^F-FDG and ^68^Ga-pentixafor PET/CT scans on two consecutive days. The nuclear medicine physician performed imaging 63 min after intravenous injection of 5.7 mCi of ^18^F-FDG. On the following day, PET/CT was acquired 30 min after intravenous injection of 3.4 mCi (125.8 MBq) of ^68^Ga-pentixafor.

**Figure 1:**
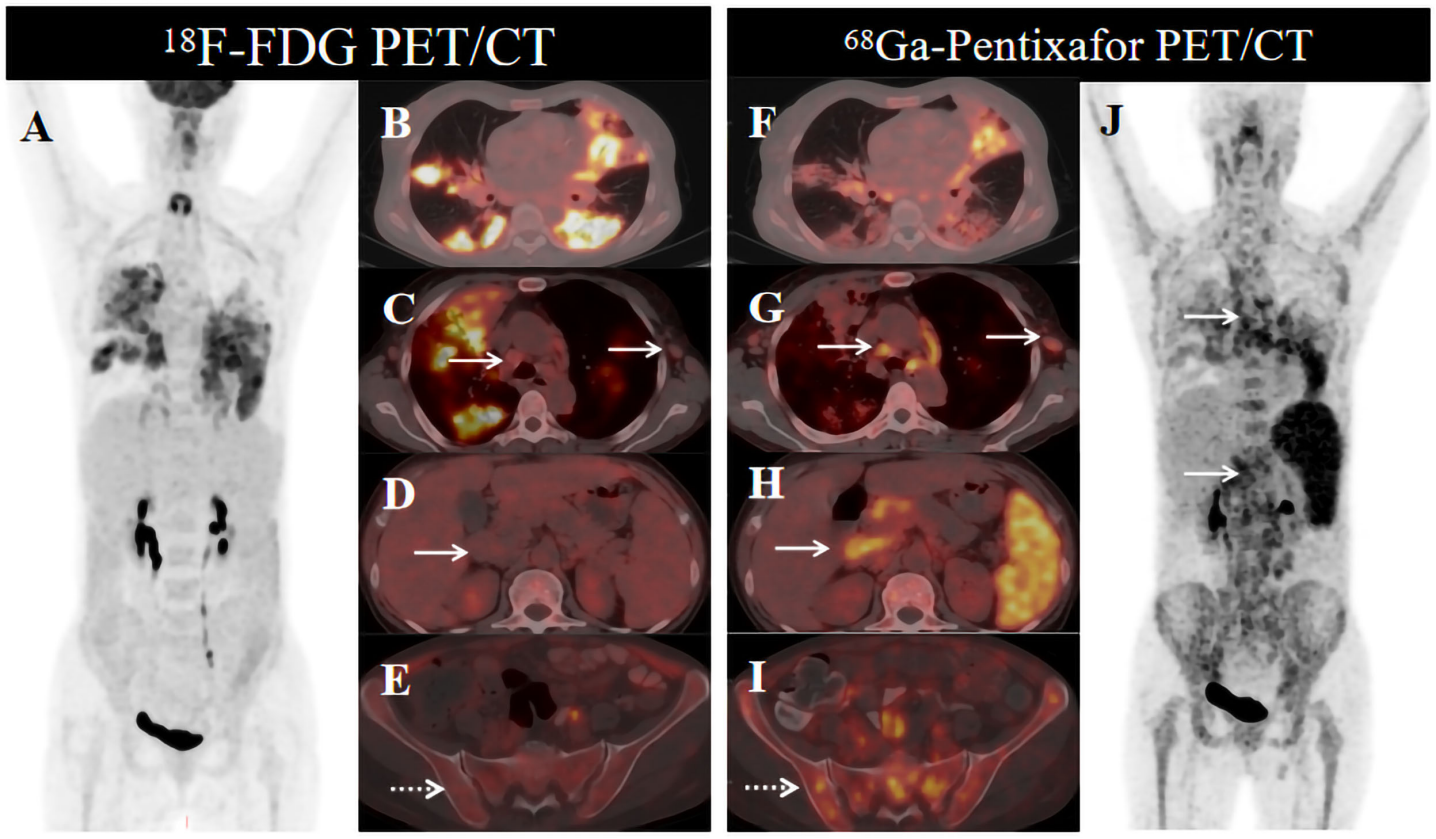
^18^F-FDG and ^68^Ga-Pentixafor PET/CT images of a 60-year-old female with CLL/SLL. ^18^F-FDG PET/CT maximum intensity projection (MIP, **A**) image shows bilateral patchy pulmonary hypermetabolism **(B)**; no significant hypermetabolism in enlarged mediastinal (**C**, white arrows), retroperitoneal (**D**, white arrows) lymph nodes or systemic bone marrow (**E**, dotted arrow). ^68^Ga-Pentixafor PET/CT MIP **(J)** image shows mild pulmonary uptake **(F)**, with markedly increased uptake in the above-mentioned lymph nodes (**G, H**, white arrows) and systemic bone marrow (**I**, dotted arrow).

**Figure 2:**
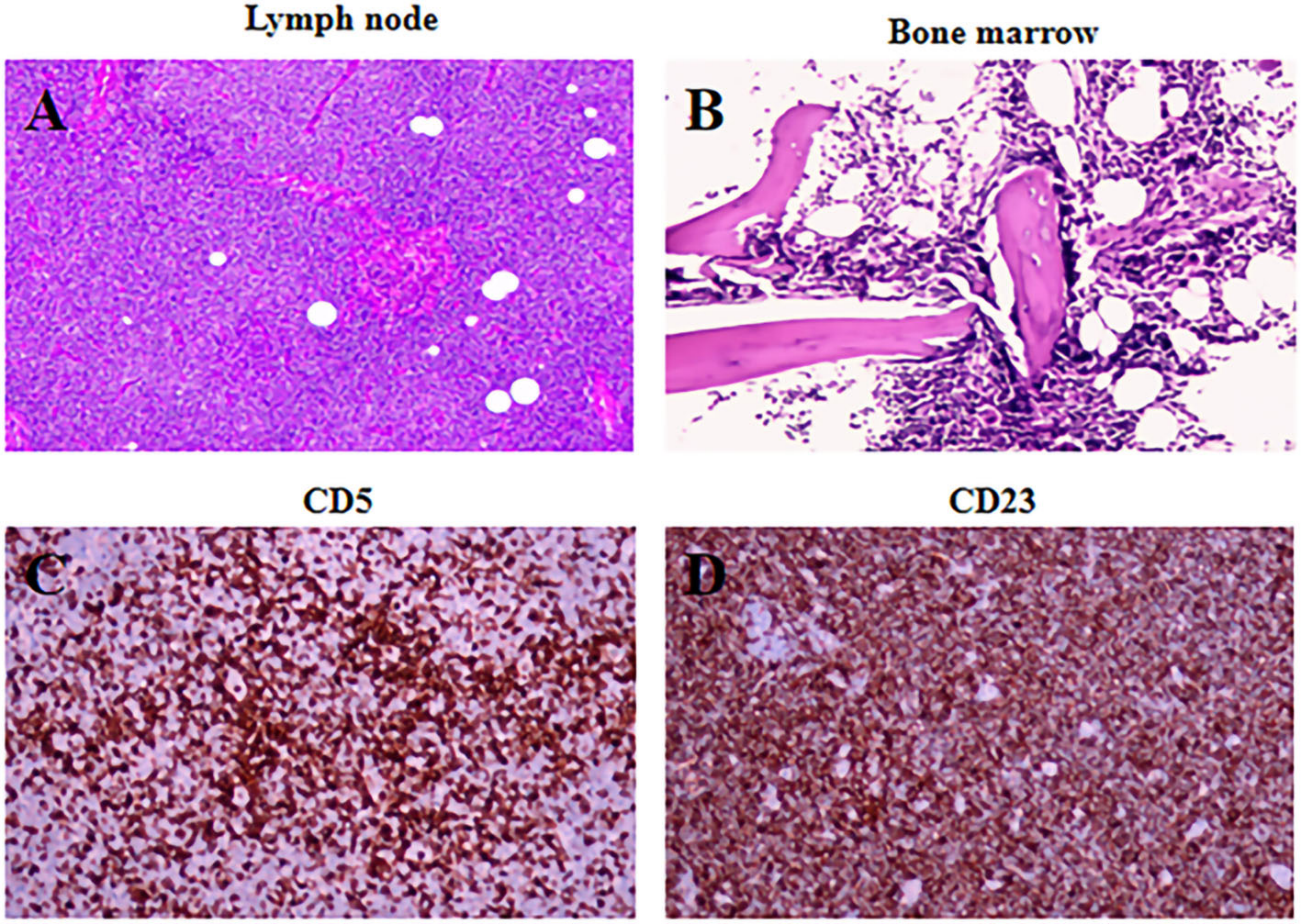
Lymph node **(A)** and bone marrow **(B)** sections with pathological microscopic imaging and immunohistochemistry expression of CD5 **(C)**, CD23 **(D)**.

To clarify the diagnosis, the patient underwent sequential molecular imaging with ^18^F-FDG PET/CT, followed by ^68^Ga-Pentixafor PET/CT the next day. The PET/CT images are shown in **Figure 1**. ^18^F-FDG PET/CT demonstrated multiple patchy hypermetabolic foci in the bilateral lungs, with maximum standardized uptake value (SUV_max_) of 5.6–7.1 (**Figures 1A, B**, maximum intensity projection (MIP) and fusion, respectively). Notably, ^18^F-FDG PET/CT showed no hypermetabolism in the multiple enlarged lymph nodes above and below the diaphragm (**Figures 1C, D**, fusion, white arrows) or in the systemic bone marrow (**Figures 1A, E**, MIP, fusion, dotted arrow). In contrast, ^68^Ga-pentixafor PET/CT revealed mild uptake in both lungs, with SUV_max_ of 1.4–4.7 (**Figures 1F, J**, fusion, MIP). Multiple hypermetabolic lymph nodes were observed above and below the diaphragm, with SUV_max_ ranging from 3.5 to 8.7 (**Figures 1G, H, J**, MIP, fusion, solid arrows). Diffuse skeletal uptake was noted in the axial skeleton and proximal appendicular bones, with SUV_max_ between 2.3 and 3.7 (**Figures 1I, J**, fusion, MIP, dotted arrow). Pathological examination revealed effacement of the lymph node architecture by diffuse small lymphocyte proliferation and increased lymphoid infiltrates in the bone marrow (**Figures 2A, B**). Immunohistochemical analysis demonstrated positivity for CD5 and CD23 (**Figures 2C, D**); focal positivity for cyclin D1; and negativity for CD10, BCL-6, and SOX11, consistent with a diagnosis of CLL/SLL with clinical stage (Binet stage C, Rai stage III). The pulmonary lesions regressed following antibiotic therapy, accompanied by symptomatic improvement, suggesting a diagnosis of pulmonary infection. After receiving zanubrutinib targeted therapy, the volume of the lymph node lesions in this patient was significantly reduced compared with the baseline level. The patient is currently under regular follow-up.

## Discussion

CLL and SLL are clinicopathologic variants of the same disease, derived from clonal mature B cells. The key difference is the predominant anatomical distribution. SLL primarily involves lymph nodes, while CLL affects the bone marrow and peripheral blood (2, 4, 5). The tumor cells typically express surface markers such as CD5 and CD23 (6). The diagnosis of CLL/SLL is primarily established based on peripheral blood and bone marrow findings, typically demonstrating monoclonal B-cell lymphocytosis in the blood and lymphocytic infiltration in the marrow. Immunophenotypic analysis is essential for confirmation. The clinical manifestations are highly heterogeneous, ranging from being asymptomatic to presenting with painless lymphadenopathy, hepatosplenomegaly, and bone marrow infiltration. The natural history of the disease varies considerably: some patients exhibit an indolent disease course and may not require treatment for many years, whereas others experience rapid progression and necessitate prompt therapeutic intervention (7, 8).

With low proliferative activity (Ki-67 < 20%) and typically low uptake on ^18^F-FDG PET, CLL/SLL shows lower ^18^F-FDG PET/CT sensitivity than aggressive lymphomas (1, 9–11). ^18^F-FDG PET/CT is a valuable modality for identifying complications in CLL/SLL, including Richter transformation (RT) and second malignancies (SMs). RT denotes an aggressive conversion to diffuse large B-cell lymphoma (DLBCL), whereas SMs indicate an elevated risk of secondary neoplasms. Notably, PET/CT demonstrates high negative predictive value in excluding RT (12).

C–X–C chemokine receptor type 4 (CXCR4) is aberrantly overexpressed in the majority of lymphoma cells, including those of CLL/SLL, which is a key chemokine receptor that promotes B-cell migration and survival. This receptor guides CLL/SLL cells to migrate and remain in these microenvironments by binding to its ligand C–X–C motif chemokine ligand 12 (CXCL12) secreted by the bone marrow and lymph node microenvironments, further obtaining protection to evade killing by chemotherapy drugs and directly promoting the proliferation of tumor cells (2, 13, 14).

^68^Ga-pentixafor exhibits specific binding affinity for CXCR4, enabling functional imaging of its expression levels, thereby facilitating the identification of lesions and determination of the disease extent. It also aids in distinguishing lymphoma from other conditions such as infection and inflammation. In addition, ^68^Ga-pentixafor PET/CT complements ^18^F-FDG PET/CT, particularly in lymphoma subtypes with low ^18^F-FDG avidity, where the sensitivity of ^18^F-FDG PET/CT is reduced. In such cases, ^68^Ga-pentixafor PET/CT may provide additional diagnostic information.

^68^Ga-pentixafor PET/CT shows potential for the early diagnosis of CLL/SLL, particularly in early-stage disease or in cases with low FDG avidity. It is also valuable for assessing the tumor burden, including lymphadenopathy, splenomegaly, and bone marrow infiltration. Consequently, ^68^Ga-pentixafor PET/CT offers enhanced sensitivity in detecting lymphoma lesions and improved accuracy in evaluating disease involvement (15).

A high CXCR4 expression is associated with more aggressive disease progression and inferior treatment response, serving as a predictor of poor prognosis in patients with CLL/SLL. ^68^Ga-pentixafor PET/CT allows noninvasive visualization of the expression distribution of CXCR4 and can assist in assessing prognostic risk. ^68^Ga-pentixafor PET/CT can be used to guide treatment selection for CLL/SLL—for instance, by identifying patients with high CXCR4 expression who may benefit from targeted therapies against CXCR4. Furthermore, ^68^Ga-pentixafor PET/CT enables the assessment of tumor cell viability and distribution by detecting changes in the expression levels of the CXCR4 receptor. This technique is valuable for evaluating treatment response and monitoring disease progression, where a post-treatment reduction in CXCR4 expression may indicate a favorable therapeutic outcome (16, 17). In summary, ^68^Ga-pentixafor demonstrates significant potential advantages in the diagnosis and management of CLL/SLL. However, reports on ^68^Ga-pentixafor PET/CT for diagnosing CLL/SLL remain scarce. Existing studies are limited to small cohorts, with the majority focusing solely on bone marrow and describing marked ^68^Ga-pentixafor uptake in this site (18). There is a lack of large-scale studies with comprehensive assessment of nodal involvement. In the present case, we observed prominent uptake of ^68^Ga-pentixafor in nodal and splenic lesions of patients with CLL/SLL. To our knowledge, the avidity of ^68^Ga-pentixafor in nodal and splenic lesions has been documented in CLL/SLL, and the present case further validates and expands on this finding (17). Future studies should further explore the role of ^68^Ga-pentixafor PET/CT in guiding CXCR4-targeted treatment strategies for patients with CLL/SLL. The expression levels of CXCR4 may vary across individual patients and disease stages, and further data are required to support its clinical interpretation.

## Conclusions

In this case, ^68^Ga-pentixafor PET/CT demonstrated superior uptake in the lymph nodes, bone marrow, and spleen compared with ^18^F-FDG PET/CT, enabling more accurate lesion localization and disease extent evaluation, thereby providing comprehensive information for diagnosis and staging. Our study not only aligns with the limited data on bone marrow uptake but also expands the findings to include nodal and splenic involvement. However, this necessitates large-scale clinical trials to confirm the advantages of ^68^Ga-pentixafor PET/CT in the diagnosis and treatment of CLL/SLL.

## Data Availability

The original contributions presented in the study are included in the article/supplementary material. Further inquiries can be directed to the corresponding authors.
